# The complete chloroplast genome sequence of Tartary Buckwheat Cultivar Miqiao 1(*Fagopyrum tataricum Gaertn.*)

**DOI:** 10.1080/23802359.2016.1197056

**Published:** 2016-08-22

**Authors:** Moyang Liu, Tianrun Zheng, Zhaotang Ma, Dong Wang, Tao Wang, Rong Sun, Zhoufeng He, Jiyan Peng, Hui Chen

**Affiliations:** College of Life Science, Sichuan Agricultural University, Ya’an, Sichuan, China

**Keywords:** Complete chloroplast genome, medicinal plant, Tartary Buckwheat Cultivar Miqiao 1

## Abstract

Chloroplasts (cp) are indispensable organelles in plant cells that perform photosynthesis amongst other functions, including producing pigments, synthesizing sugars and certain amino acids. The complete chloroplast genome of Tartary Buckwheat Cultivar Miqiao 1 is sequenced in this study. Miqiao 1 is currently the only Tartary Buckwheat variety that can produce the whole nutritive Tartary Buckwheat Rice by hulling. The Miqiao 1 cp genome size is 159,272 bp in length, including 79 protein-coding genes, 30 tRNA genes and 4 rRNA genes. A pair of inverted repeats (IRs) of 30,817 bp were disconnected by a large single copy (LSC) of 84,397 bp and a small single copy (SSC) of 13,241 bp. The cp genome with 37.9% GC content contained 113 annotated genes.

Chloroplasts (cps) are indispensable plant cell organelles that conduct photosynthesis in the presence of daylight. The chloroplast genome in land plants is a circular molecule ranging in size from 76 to 217 kb, with a highly conserved structure of two copies of large inverted repeats (IR) detached by small (SSC) and large (LSC) single-copy regions (Dane et al. 2015). Characteristics of the cp genome have been used comprehensively as a tool to investigate evolutionary relationships and molecular phylogenetic of seed plants (Jansen et al. 2011).

Buckwheat (*Fagopyrum* species) which belongs to *Polygonaceae*, a member of knotgrass, is an annual herbaceous plant. Buckwheat is classified into 20 species. Geographically, it is principally centred in the Eurasian region and grown in the highlands. Further, it is an extensively cultivated multipurpose crop (Kump & Javornik 1996; Ohsako et al. 2002). Tartary buckwheat is an exceptionally opulent source of rutin compared to common buckwheat. Rutin assists moderate diabetes, high blood pressure and intravascular cholesterol (Fabjan et al. 2003). It is also reported to have a decisive role in pharmaceutical research (Kim & Kim 2004). Miqiao 1 is currently the Tartary Buckwheat variety that can produce the Whole Nutritive Tartary Buckwheat Rice by hulling. Recently, the cp genome sequence of some plants have been reported, but medicinal plants are rarely reported (Dane et al. 2015). Three species of *Fagopyrum Mill* have been reported recently (Logacheva et al. 2008; Cho et al. 2015; Yang et al. 2015).

The genomic DNA was extracted from the leaves of *Fagopyrum tataricum Gaertn.* cultivated in the field of College of Life Science, Sichuan Agricultural University, Ya’an, China. This study reports the sequencing of the Miqiao 1. The whole cp genome was sequenced using HiSeq 4000 PE150 Illumina (Biocode Biotech Co., Beijing, China) and assembled into the complete cp genome using Sequencher version 4.10 (Bankevich et al. 2012). DualOrganellarGenoMe Annotator (Wyman et al. 2004) was used to annotate the cp genome with BLASTX and BLASTN identifying the location of encoding genes and RNA (Altschul et al. 1997). The complete genome sequence was submitted to GenBank under the accession number KX085498.

The total size of complete cp genome was 159,272 bp with 37.9% GC content, consisting of one LSC (84,397 bp), one SSC (13,241 bp) and two IRs (30,817 bp). The GC content of the IRs (41.3%) was higher than that of LSC and SSC (36.2% and 32.8%, respectively). A total of 113 genes were successfully annotated, including 79 protein-coding genes, 30 tRNA genes and 4 rRNA genes. There were 17 intron-containing genes, all of which were single-intron genes except for *ycf3* and *clpP* with two introns separately. *rpsl2* was a reverse-splicing gene, of which the 5′ end exon located in the LSC region and 3′ end exon was in the IR region. The phylogenetic relationship of the Miqiao 1 was deduced by comparing it with other nine chloroplast genomes downloaded from the GenBank (Figure 1).

**Figure 1.**
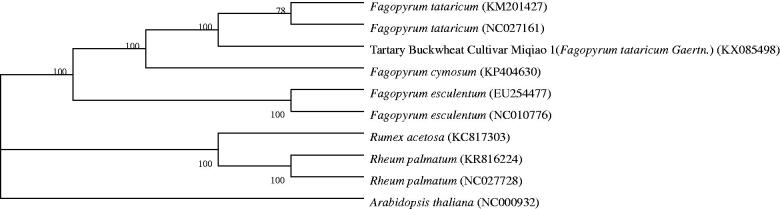
ML tree based on Tartary Buckwheat Cultivar Miqiao 1 chloroplast genomic sequence compared with 9 sequences obtained from NCBI.
